# Case of Hemorrhagic Variola

**Published:** 1872-07

**Authors:** A. Chenoweth

**Affiliations:** Cook County Hospital


ARTICLE II. — *Case of Hemorrhagic Variola.* By A. CHENOWETH, M.D., Cook County Hospital.

H. R., of Germany, aged 26 years; by occupation a carpenter; spent the early part of his life on a farm. He afterward entered the army, where he remained for four years, taking part in the late Franco-German war. His diet during this time was not of the kind most conducive to health, consisting principally of salt pork. His health had been comparatively good, with the exception of a convulsion which he experienced about seven years ago, at which time the blood gushed from his nostrils and eyes. Having arrived in this country about five weeks previous to his last illness, he again resumed his occupation. Says that he was at work on the 18th of May, came home at noon feeling unwell, complained of pain in his head, chest, throat and bowels, face flushed; a physician was called in and prescribed, but the patient being delirious during the night took all of his medicine, regardless of time or dose. The patient commenced expectorating blood on the 20th. On the 21st of the month, three days after the first attack, he was admitted to the hospital. Pulse 126, respirations 44, temperature 103; tongue covered with a thick brown coating, fissured in the centre and marked by purpuric spots at the edge; the breath of an offensive, sanguineous odor; small, slightly elevated patches appeared all over the trunk and limbs, while the anterior portion of the chest and outer portion of the forearms presented a uniform red aspect.

May 22d, 9 o'clock, A. M. Pulse 120, respirations 30, temperature 102. Patient slept but little during the night; expectorated about one pint of blood during the night; stools frequent and bloody, urine scanty, but of a deep red hue; breath very offensive. Prescribed R. Aromatic Sulph. Acid, dr. ij; Aqua, oz. viij. S. Tablespoonful every two hours. R. Acid Gallic, grs. xlv; Syr. Acaciæ, oz. ij. S. Teaspoonful every three hours.

One o'clock, P. M. Our attention was drawn to the characteristic variolous eruption which appeared on the limbs and chest, with three or four papules on the face; owing to the redness of the surface and the color of the variolous eruption, attention had not been directed to the papula characteristic of variola previous to this time, some of which had now become umbilicated. The skin, even where of a uniform red color, appeared elevated. The patient, notwithstanding the painful deglutition, took three pints of milk during the last twenty-four hours. Patient makes no complaint of pain in the back, as is usual in the genuine uncomplicated small-pox.

Here I lost sight of the case, as it was deemed necessary that he should be removed to the small-pox hospital, but through the kindness of the physician in charge, Dr. Cogne, I was put in possession of the subsequent history.

May 23rd. Patient still expectorating blood. Numerous unhealthy vesicles appearing all over the body, and although iron, quinine, milk-punch, beef-tea and other valued remedies were administered, the character of the eruption remained unchanged.

May 24th. Hemorrhage continued; pulse 130 and very weak; respiration hurried and oppressed; breath horribly offensive; skin hot and dry, becoming livid; vesicles numerous; bowels and kidneys acting freely.

May 25th. All the bad symptoms aggravated; the skin now nearly black; the vesicles appearing of an ashy color; blood oozing from nearly all the mucous surfaces; respiration difficult; pulse very weak, and extremities cold.

The patient remained in this condition for a few hours when death took place.

